# Vehicle Interaction Behavior Prediction with Self-Attention

**DOI:** 10.3390/s22020429

**Published:** 2022-01-07

**Authors:** Linhui Li, Xin Sui, Jing Lian, Fengning Yu, Yafu Zhou

**Affiliations:** Key Laboratory of Energy Conservation and New Energy Vehicle Power Control and Vehicle Technology, School of Automotive Engineering, Faculty of Vehicle Engineering and Mechanics, Dalian University of Technology, Dalian 116081, China; lilinhui@dlut.edu.cn (L.L.); suixin1998@mail.dlut.edu.cn (X.S.); yfn19941208@mail.dlut.edu.cn (F.Y.); dlzyf@dlut.edu.cn (Y.Z.)

**Keywords:** vehicle interaction behavior prediction, self-attention, vehicle cluster, end-to-end prediction, class imbalance

## Abstract

The structured road is a scene with high interaction between vehicles, but due to the high uncertainty of behavior, the prediction of vehicle interaction behavior is still a challenge. This prediction is significant for controlling the ego-vehicle. We propose an interaction behavior prediction model based on vehicle cluster (VC) by self-attention (VC-Attention) to improve the prediction performance. Firstly, a five-vehicle based cluster structure is designed to extract the interactive features between ego-vehicle and target vehicle, such as Deceleration Rate to Avoid a Crash (DRAC) and the lane gap. In addition, the proposed model utilizes the sliding window algorithm to extract VC behavior information. Then the temporal characteristics of the three interactive features mentioned above will be caught by two layers of self-attention encoder with six heads respectively. Finally, target vehicle’s future behavior will be predicted by a sub-network consists of a fully connected layer and SoftMax module. The experimental results show that this method has achieved accuracy, precision, recall, and F1 score of more than 92% and time to event of 2.9 s on a Next Generation Simulation (NGSIM) dataset. It accurately predicts the interactive behaviors in class-imbalance prediction and adapts to various driving scenarios.

## 1. Introduction

In the traffic scene, the perception technology of intelligent vehicles can help their vehicles perceive the complex environment. The Society of Automotive Engineers (SAE) has defined six different levels of driver assistance technology [1]. Higher-level autonomous driving technology requires better environment perception capabilities, not only to detect the relevant objectives of the current environment but also to predict the associated potential risks of the ego-vehicle. For the ego-vehicle, both its behavior and surrounding objects may cause potential risks. The prediction for the ego-vehicle is due to the driver’s predicted intention, which is based on the vehicle manipulation signal of the On-Board Diagnostic (OBD) and the in-vehicle camera. It also considers operating information and personal driving style [2]. The objects around the ego-vehicle are composed of static objects and dynamic objects. Real-time changes of dynamic objects require not only detecting the object’s position but also predicting the intention of the object. As the most common traffic participants in the driving environment, the surrounding vehicles can reduce the transfer of control rights of the ego-vehicle system by predicting the change of the right of the way relative to the ego-vehicle at an early stage [3].

There are different vehicle flows at different times in the traffic scene. If similar time series occur when the traffic data is normally similar, future traffic value is likely to be similar [4]. Therefore, the three traffic flow conditions, under the condition build-up, or the transition between unconstrained and conditioned conditions, and full condition during the peak period, cover various road environment data types. At the same time, traffic planning is committed to predicting future traffic conditions and using the information to optimize vehicle routes to reduce traffic congestion and improve traffic mobility [5]. Therefore, in the actual traffic environment, human drivers will also tend to avoid congestion. The prediction of vehicle interaction behavior needs to adapt to different traffic flow conditions.

In response to the above problems, this paper proposes a novel prediction model, focusing on the target vehicle that significantly impacts the ego-vehicle. A five-vehicle cluster is proposed to extract interactive information. In other studies, time-series information is also used as the prediction input. To avoid recursion, achieve parallel computing, and reduce the performance degradation caused by long-term dependence, this paper uses the multi-head self-attention encoder to understand timing information. Furthermore, this paper proposes a VC-Attention to improve vehicle interaction behavior prediction.

The remainder of this paper is organized as follows: Section 2 introduces the related works and existing problems of vehicle behavior prediction. Section 3 briefly introduces the construction of a five-vehicle cluster, the prediction model based on self-attention encoder, and the extraction of interactive features and the optimization of prediction models. Section 4 presents the experiments and an analysis of the results. The paper ends with our conclusions.

## 2. Related Works

The field of advanced driver assistance systems (ADAS) has matured, and vehicle interaction behavior prediction is essential [6]. Two sensor signals are widely used in lane change prediction of surrounding vehicles, including driver behaviors and traffic context [7]. For instance, deep recurrent neural networks with driving maneuver behaviors and traffic environment context predict drivers’ imminent maneuvers [8]. The traffic context is similar to the observation information received by radar and camera. The intelligent cruise control in the advanced driving assistance system uses the surround-view surveillance camera to realize the blind-spot cut-in warning of the surrounding vehicles by combining with the tire detection vehicle [9]. The novel vision data is used to predict modified lane change prediction with object detector in multi-layer perceptron algorithms [10]. Based on the video information, the Two-Stream Convolutional Networks, the Two-Stream Inflated 3D Convolutional Networks, the Spatiotemporal Multiplier Networks, and the SlowFast Networks are used to identify and predict interactive behaviors by analyzing different size areas around the vehicle [11]. The above method has insufficient advance time in predicting vehicle right-of-way change, which poses a significant challenge to implementing corresponding feedback for the ego-vehicle.

As for implementing lane changing actions, driver motivation, such as extracting steering wheel angle, angular velocity, angular acceleration, is also used as a feature input to identify the driver’s intention to change lanes in the early stage [12]. For instance, driver behavior recognition based camera information is used in manifold learning to predict lane-change behavior [13]. Further considering the driving habits, based on the Gaussian distribution of the hybrid HMM, a driver’s lane changing intention recognition model is constructed for each driver [14]. However, driver behavior identification may interfere with driver behavior and even violate privacy.

There is a certain equivalence between vehicles’ lane change intention prediction and driver lane change intention recognition. Because of the current sensing technology, the ego-vehicle cannot directly detect driver behavior of the target vehicle but can only obtain traffic context. The driver’s lane change intention is also affected by traffic context, such as the slow leading vehicle, return lane, and merging vehicle, which can be used as input for lane change reasoning [15]. Therefore, the vehicle interaction information that affects the driver’s intention is used to predict the change of vehicle right-of-way. Using the traffic context, the Support Vector Machine (SVM), Bayesian Net-work (BN), Logistic Regression (LR) [16], Artificial Neural Network (ANN) [17], and eXtrem Gradient Boosting (XGBoost) [18] present good performance in lane change prediction. As for traffic context, there is a method of predicting the driver’s intention through uncertainty multi-modal trajectory prediction with trajectory history [19], using the Gaussian mixture model to identify and predict lane change intentions through the context information on four adjacent vehicles and the target vehicle [20]. To tackle time series prediction problems, an intention inference model based on recurrent neural networks (RNNs) is proposed [21]. In highway interweaving areas, the long-short-term memory network (LSTM) is used to predict the future trajectory of the lane-changing vehicle [22].

To process timing information, a RNN model is proposed such that the calculation of this time step depends on the information of the previous time step. To solve the gradient disappearance and obtain a longer-term information connection, memory, update and forget gates are proposed in LSTM to capture deeper connections between timing information. To avoid recursion, achieve parallel computing, and reduce the performance degradation caused by long-term dependence, the timing information is processed through the self-attention layer to obtain the previous time step information. Moreover, information on the vehicle cluster is needed to obtain traffic context.

## 3. Model and Proposed Methods

**Figure 1:**
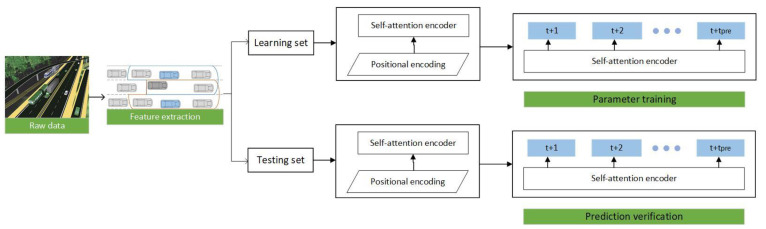
Framework of proposed prediction approach.

The core of our system is a self-attention encoder with a two-layer structure, which fully considers the predictive features and the relevance of continuous maneuvers, as shown in Figure 1.

Firstly, this paper determines the current lane and the target lane by the ego-vehicle and the target vehicle to divide the five-vehicle clusters. Secondly, the vehicle cluster information is extracted, which includes the interactive features of the target vehicle relative to the ego-vehicle, the surrounding vehicles relative to the target vehicle, and the vehicle cluster. Then, based on the time series information, the interactive features of the vehicle cluster are extracted through the sliding window. Finally, the batch normalized features are input into the self-attention encoder for subsequent vehicle right-of-way change prediction.

### 3.1. Intent Prediction Model

**Figure 2:**
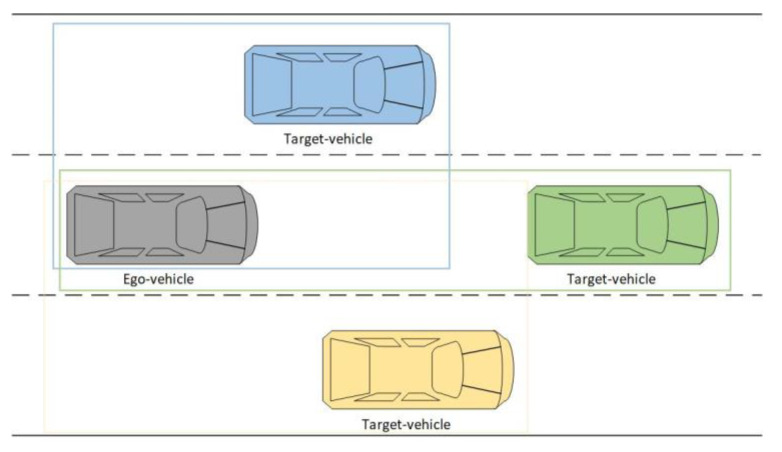
Diagram of case division.

This paper aims to establish a lane change prediction model for surrounding vehicles, focusing on the target vehicle that significantly impacts the ego-vehicle. In the general lane change scenario, as shown in Figure 2, the gray vehicle is the ego-vehicle, and the blue, green, and yellow vehicles are target vehicles driving in the right and left adjacent lanes and the current lane, respectively. The scope of target vehicles is vehicles driving in the adjacent lanes between the ego-vehicle and ego-vehicle’s front vehicle and the front vehicle, as those target vehicles may change lanes and the change of right-of-way. This paper establishes lane change prediction models for cut-in or cut-out of target vehicles. The vehicle clusters around the ego-vehicle are divided into three five-vehicle clusters based on the three target vehicles in the front left, front, and front right.

**Figure 3:**
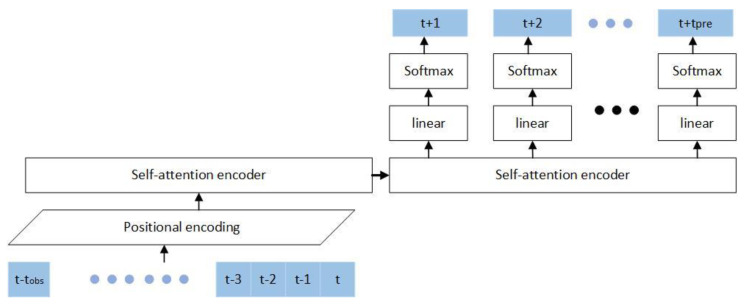
The structure of the VC-Attention.

Under the driving situation, if there is no other vehicle blocking, lane-changing behavior will be avoided as much as possible. If the front vehicle speed is too slow or the driving space in the adjacent lane is sufficient, the timing of the lane change will be judged according to the state of the surrounding vehicles, and the lane change behavior will occur [23]. Our research assumes that sensors of the ego-vehicle could detect the state of the vehicle cluster in their detection range. The interaction between the cluster and the self-vehicle, the cluster and the target vehicle, and the clusters are used as time series information to predict the intention of the target vehicle lane changing. At any time t, extract the interactive features of the five-vehicle cluster, take $t\sim t-t_{obs}$ as the observation frames, and $t+1\sim t+t_{pre}$ the prediction frames. The prediction result is a time series one-hot coding matrix, which predicts three intentions of right-of-way encroachment (cut-in), right-of-way release (cut-out), and no change of right-of-way (no-cut). The primary neural network structure of this article is shown in Figure 3.

The structure proposed in this paper includes the feature extraction of vehicle clusters with ego-vehicle and target vehicle as to the main body, and the processed features obtain time-series information through pose embedding, which is coded through two self-attention coding layers. The vehicle cluster information is encoded and inputs into the decoding layer, which uses an end-to-end prediction method to predict vehicle intentions to avoid error accumulation. If the prediction information relies on the previous frame, the prediction error of the previous frame cumulatively affects the prediction results of all subsequent frames.

### 3.2. Self-Attention Encoder

The processing of time-series information usually uses Recurrent Neural Network (RNN), so that the calculation of this time step depends on the information of the previous time step. To solve the gradient disappearance of RNN, Gated Recurrent Unit (GRU) is introduced to capture deep connections through memory gates and update gates. To obtain a longer-term information connection, a forgetting gate is introduced based on GRU to capture deeper connections between timing information through LSTM. The cyclic neural network processes the timing information serially. To avoid recursion, achieve parallel computing, and reduce the performance degradation caused by long-term dependence, the timing information is processed through the self-attention layer to obtain the previous time step information.

The self-attention mechanism disassembles the timing information so that the input information has no timing dependence, which means the near-frame information and the far-frame information have the same influence on the prediction. Therefore, it is necessary to distinguish the near and far frame information by adding time sequence coding [24], as shown in Equation (1).

(1) $$X_{embedding}=X_{embedding}+X_{pose},$$

where $X_{pose}$ is position information, and $X_{embedding}$ is the information before and after positional encoding. Since the length of the input time sequence is small, the time sequence information is encoded into the input information through self-learning positional encoding to add time sequence dependence.

Attention is a mapping relationship between a set of key-value pairs, for which the corresponding value can be obtained by querying the corresponding key. The traditional attention mechanism forms the corresponding relationship between query value and key-value pair through the corresponding relationship between input and output [25], while self-attention is to construct the mapping relationship between the query and key-value pair based on the relationship between input sequence information. Obtain the output value through weighted calculation. Multiply the input sequence information with different conversion matrices to obtain query values and key-value pairs [26], as shown in Equation (2).

(2) $$\begin{array}{c} Q=X\times W_{q}\\ K=X\times W_{k}\\ V=X\times W_{v} \end{array}$$

where Q is the query vector, K is the key vector of the correlation between the queried information and other information, and V is the value vector of the queried vector. Through the query vector and the key vector of the correlation between the queried information and other information, the scaled inner product is used to obtain the correlation measure between the input information, and the dot product self-attention is obtained, as shown in Equation (3).

(3) $$\alpha_{i,j}=\frac{Q_{i}\cdot K_{j}}{\sqrt{d}},$$

where $\alpha_{i,j}$ is the dot product self-attention, and d is the dimension of the key vector. The scaled dot-product attention is the dot product self-attention weight of each queried information relative to the query vector. This method selectively obtains branch information through the information relevance flag. If $\hat{\alpha }=0$, the branch information is not considered, as shown in Equation (4).

(4) $${\hat{\alpha }}_{i,j}=\frac{\exp\alpha_{i,j}}{\sum_{p}\exp\alpha_{i,p}},$$

where ${\hat{\alpha }}_{i,j}$ is the scaled dot-product attention. It can be equivalent to the weighted summation of the value vector of the query vector through the softmax layer as the information relevance flag to obtain the timing feature, as shown in Equation (5).

(5) $$Attention(Q,K,V)=softmax(\frac{QK^{T}}{\sqrt{d}})V,$$

where Attention is the result of self-attention encoder. Dot product self-attention is implemented using highly optimized matrix multiplication codes, saving calculation time and space.

**Figure 4:**
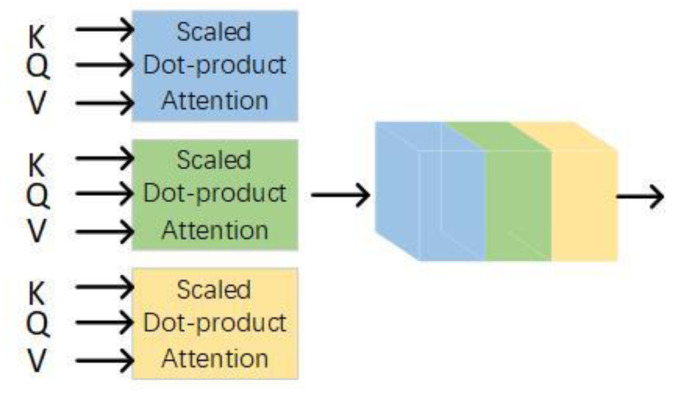
The structure of the multi-head attention.

A multi-head attention mechanism extracts multiple independent semantics to prevent over-fitting. Use different linear projections to generate query vectors, key vectors, and value vectors, perform dot product attention in parallel, and then connect them for re-projection, as shown in Figure 4. Different linear projections focus on different subspace information, and the total computational cost does not increase. After each layer, the residual connection is used to prevent network degradation, and the activation value of each layer is normalized.

In layer normalization, the input of neurons in the same layer has the same mean and variance, and different input samples have different mean and variance. Compared with batch normalization, it is necessary to calculate and save the mean and variance of a certain layer of neural network batch. Statistical information is more suitable for time series models with depth changes. Batch normalization is shown in Equation (6).

(6) $$\begin{array}{ll} \mu^{l} & =\frac{l}{H}\sum_{i=1}^{H}a_{i}^{l}\\ \sigma^{l} & =\sqrt{\frac{l}{H}\sum_{i=1}^{H}(a_{i}^{l}-\mu^{l}{)}^{2}}\\ a_{i}^{-l} & =\frac{g^{l}}{\sigma^{l}}\cdot (a_{i}^{l}-\mu^{l})+b \end{array}$$

where l is the lth hidden layer, H is the number of nodes in this layer, and a is the value of a certain node before activation, that is, a=wx+b, where w is gain parameter and b is bias parameter, which can be included in training and training with a group of samples. At the same time, it contains a fully connected feedforward network as shown in Equation (7), including two linear transformations, and the first layer uses the RELU activation function.

(7) $$FNN(x)=\max(0,xW_{1}+b_{1})W_{2}+b_{2},$$

where FNN is the result of the feedforward network.

**Figure 5:**
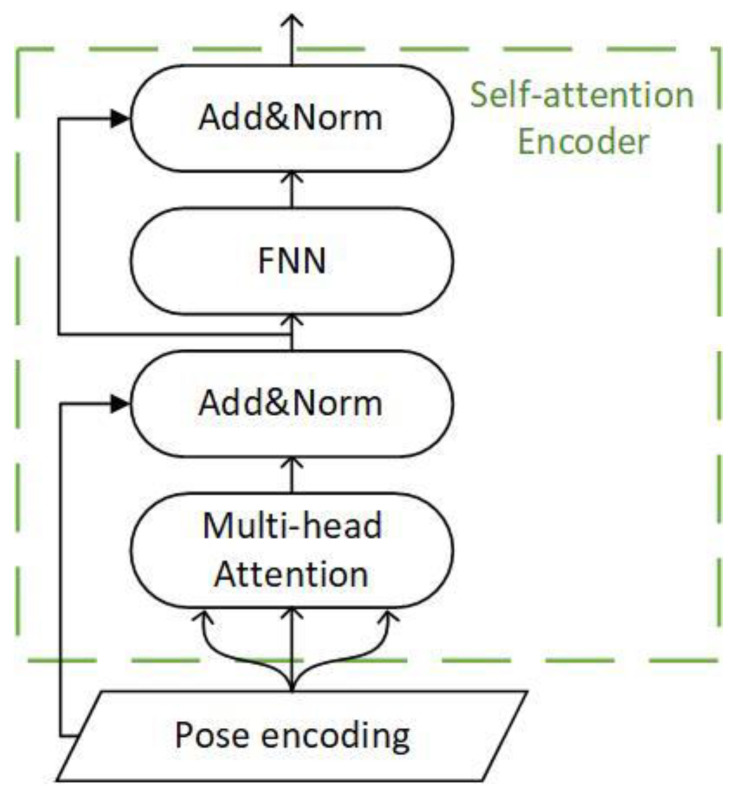
The structure of the self-attention encoder.

For the time series model, the input tokens are converted into tensors by positional encoding, and multiple independent semantics are obtained through multi-head self-attention splicing. Then the feedforward network is connected through residual connection and layer normalization, and the encoded output is obtained through residual connection and layer normalization. The whole constitutes a self-attention encoder, as shown in Figure 5.

### 3.3. Interactive Feature Extraction

**Figure 6:**
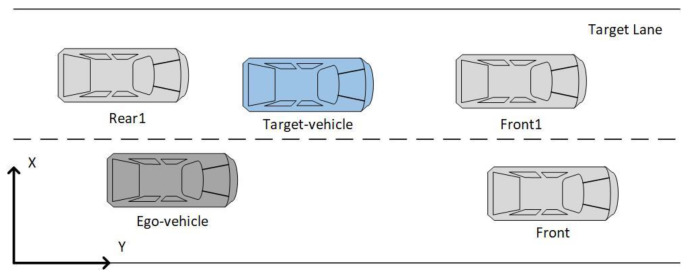
The five-vehicle cluster.

Establish a coordinate system based on the ego-vehicle, which focuses on the front vehicle of the current lane and the adjacent vehicles on both sides of the lane. Taking the front vehicle in the current lane and the adjacent vehicle in the left and right lane as the target vehicles, three-vehicle clusters focusing on the behavior of the target vehicles are constructed. The five-vehicle cluster centered on the ego-vehicle and the target vehicle also includes the front vehicle in the current lane and the front and behind vehicles in the target lane to construct a dynamic environment of two adjacent lanes shown in Figure 6. If the target vehicle and the ego-vehicle are in the same lane, the target lane is selected according to the lateral speed direction of the target vehicle. In a complex traffic environment, the change of vehicle’s trajectory coordinates cannot fully reflect the interaction between the vehicles during the lane change and their neighbors. Therefore, the interactive information between the cluster vehicles is introduced.

The surrounding five-vehicle cluster features are divided into three types: the interactive information of the target vehicle relative to the ego-vehicle, the interactive information of surrounding vehicles relative to the target vehicle, and the interactive information of the vehicle cluster. In the interactive information of surrounding vehicles relative to the target vehicle, the Deceleration Rate to Avoid a Crash (DRAC), which characterizes the driving risk, is introduced as an early warning study of dangerous driving behavior [27]. The collision avoidance deceleration refers to the minimum deceleration required by the following vehicle to match the vehicle’s speed in front of its target lane and avoid a collision. When the vehicle is ready to change lanes, it can be regarded as the potential risk degree of the vehicle and the vehicle in the same lane, the vehicle before and after the target lane, then the DRAC value of the vehicle at time t is shown in Equation (8).

(8) $$DRAC=\left\{ \begin{array}{ll} \frac{{\left(v_{fol}(t)-v_{pre}(t)\right)}^{2}}{d} & v_{fol}(t)>v_{pre}(t)\\ 0 & else \end{array}\right.,$$

where $v_{fol}\left(t\right)$ is the speed of the following vehicle at time t, $v_{pre}\left(t\right)$ is the speed of the preceding vehicle at time t, and d is the relative distance between the following vehicle and the preceding vehicle. Among the three types of interactive information, the interactive information of the target vehicle relative to the ego-vehicle and the interactive information of surrounding vehicles relative to the target vehicle include the relative speeds, relative accelerations, and relative positions. The interactive information of the vehicle cluster includes relative positions and the gaps in the lanes—the DRAC and the gap supplement the deficiencies of traditional trajectory information in the prediction. The specific interaction characteristics are shown in Table 1.

### 3.4. Model Optimization

Data normalization can effectively eliminate the influence of different indicators corresponding to different dimensions and units, as shown in Equation (9). By uniforming the range of different feature values, the gradient descent speed and the model accuracy are improved, and the gradient explosion is prevented.

(9) $$x^{*}=\frac{\left(x-\mu \right)}{\sigma },$$

where μ is the mean of the data, and σ is the standard deviation of the data. The multi-class model adopts the cross-entropy loss function, as shown in Equation (10), to judge the closeness of the actual output and the expected output, and the output value after softmax is used to simplify the cross-entropy function.

(10) $$H(p,q)=-\sum_{x}\left(p(x)\log(q(x)\right),$$

where p(x) is the true value, and q(x) is the predicted value.

**Figure 7:**
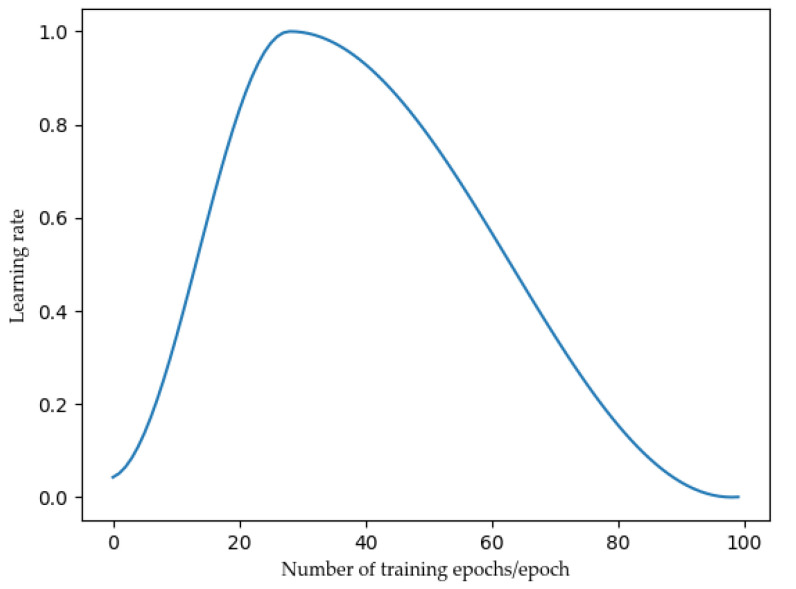
The learning rate changes with the number of iterations.

Adaptive Momentum Estimation uses momentum and root mean square based on gradient descent to improve the performance of sparse gradient problems and adjusts it according to the average value of the weight gradient. The back-propagation gradient ${\hat{d}}_{t}$ at time step t is shown in Equation (11).

(11) $$\begin{array}{cc} Vd_{t} & =\beta_{1}Vd_{t-1}+(1-\beta_{1})d_{t}\\ Sd_{t} & =\beta_{2}Sd_{t-1}+(1-\beta_{2})d_{t}^{2}\\ {\hat{d}}_{t} & =\frac{Vd_{t}/(1-\beta_{1}^{t})}{\sqrt{Sd_{t}/(1-\beta_{2}^{t})}+\varepsilon } \end{array}$$

where $d_{t}$ is the current time step gradient, $Vd_{t}$ is the one-order moments estimation of the gradient at time step t, $Sd_{t}$ is the two-order moments estimation of the gradient at time step t, ε is added to maintain numerical stability, mostly 1 × 10^−8^, $\beta_{1}$ is the exponential decay rate of the one-order moments, mostly 0.9, and $\beta_{2}$ is the exponential decay rate of the two-order moments, mostly 0.999. L2 regularization controls model complexity, reduces overfitting, adds penalty items to the original loss function, and punishes models with high complexity, as shown in Equation (12).

(12) $$\hat{J}(\omega ,x,y)=J(\omega ,x,y)+\lambda ||\omega |{|}_{2}^{2},$$

where x is the training sample, y is the training label, ω is the weight coefficient vector; λ is the control of the regularization strength parameter, which default is 0.001. To solve the obvious defect of Adam convergence proof [28], L2 regularization is combined with Adam through scaling, as shown in Equation (13), so that the weight with large gradient information will not decay like the decoupled weight, and the AdamW with decoupled weight attenuation is obtained [29].

(13) $$\begin{array}{l} d_{t}=\nabla f(\theta_{t-1})+\lambda \theta_{t-1}\\ \theta_{t}=\theta_{t-1}-\eta_{t}(\alpha {\hat{d}}_{t}+\lambda \theta_{t-1}) \end{array}$$

where $\theta_{t}$ is the parameter vector of t, $\nabla f\left(\theta_{t-1}\right)$ is $\theta_{t-1}$ corresponding gradient, λ is the control regularization strength parameter of L2 regularization, $\eta_{t}$ is the weight attenuation rate, and α is the learning rate. The learning rate adopts the one-cycle strategy, which increases from the initial learning rate to the maximum learning rate, and then decreases from the maximum learning rate to the final learning rate. The learning rate changes with training times, as shown in Figure 7, which can better match Adamw [30].

Increasing the learning rate can help the loss function value escape the saddle point. One-cycle adapting the learning rate ensures that values near the optimal learning rate are used throughout the training process [31].

## 4. Experiments and Result Analysis

### 4.1. Datasets

**Figure 8:**
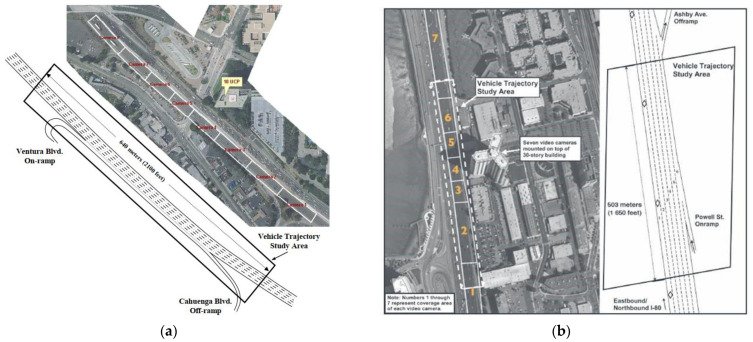
The extent of the NGSIM dataset study area. (**a**) The extent of the US101 dataset study area. (**b**) The extent of the I80 dataset study area.

This paper uses the US101 and I80 highway datasets in NGSIM, each vehicle’s position, speed, acceleration, and headway, at a frame rate of 10 fps, on US Highway 101 and Interstate Highway 80 [32]. The dataset includes vehicle trajectory data under the congestion buildup, or the transition between uncongested and congested conditions, and full congestion during the peak period. The US101 dataset is 640 m, with 5 main roads, 1 auxiliary lane, and 2 ramps, and the I-80 dataset is about 500 m, with 6 main roads and 2 ramps, as shown in Figure 8. The use of multi-road datasets can expand the amount of data, introduce the information noise of vehicle clusters in different road environments simultaneously, and improve the model’s generalization ability for different road conditions.

This paper extracts the lane-changing vehicle and its surrounding vehicle information from the NGSIM data set through a sliding window, a total of 14,552 data fragments. Based on the interactive information of the surrounding 5 vehicle clusters in the first 10 frames, the lane-change intention of the vehicles in the subsequent 10 frames is predicted. The overall data is divided into the training and test sets at 8:2. 11,643 training segments and 2909 test segments are obtained. The starting point of the lane change intention is when the vehicle’s lateral velocity is larger than 0.2 m/s in the first three consecutive frames. The endpoint of the lane change intention is when the vehicle’s lateral velocity is less than 0.2 m/s in the last three consecutive frames.

### 4.2. Experimental Settings

#### 4.2.1. Training Parameter Settings

**Figure 9:**
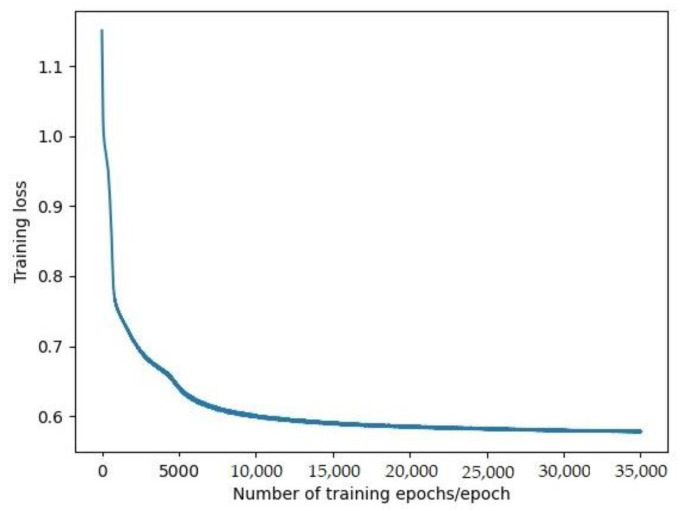
Loss during training.

In this model, the multi-headed self-attention encoder uses six heads. The feedforward neural network dimension is 128, and Relu is set as the activation function. There is a two-layer self-attention encoder when the dropout is 0.1. The input dimension is the number of observation frames, and the number of features per frame is 42. The AdamW optimization algorithm is used to decouple the weight attenuation and avoid gradient sparseness. The exponential decay rate of the one-order moment is set to 0.9, the exponential decay rate of the two-order moment is set to 0.999, and the attenuation weight is set to 0.0004. The learning rate adopts a single-cycle strategy, and its maximum learning rate is 0.001. The input dimension of the fully connected layer is 42, and the output dimension is 3. All models are trained through 35,000 iterations, and the loss results are shown in Figure 9.

**Figure 10:**
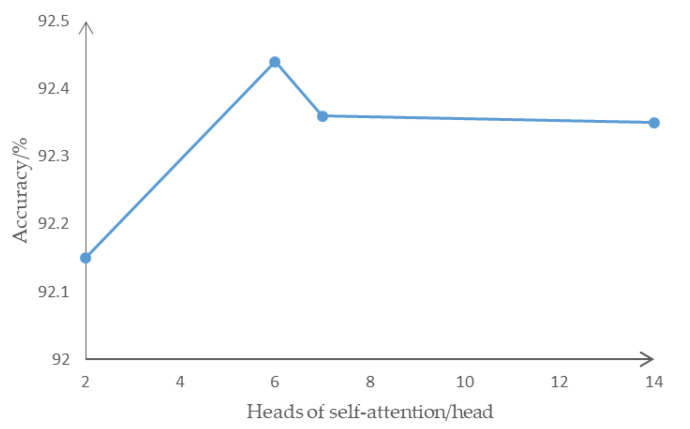
The prediction accuracy of different heads of self-attention.

After iterative training, the variance between the training set and the test set is the smallest, and the accuracy of the test set stabilizes and no longer increases. In selection of multi-head self-attention, multiple attention calculations extract multiple independent features and then integrate them. In the self-attention model with 2 to 14 heads, the prediction accuracy is shown in Figure 10.

**Figure 11:**
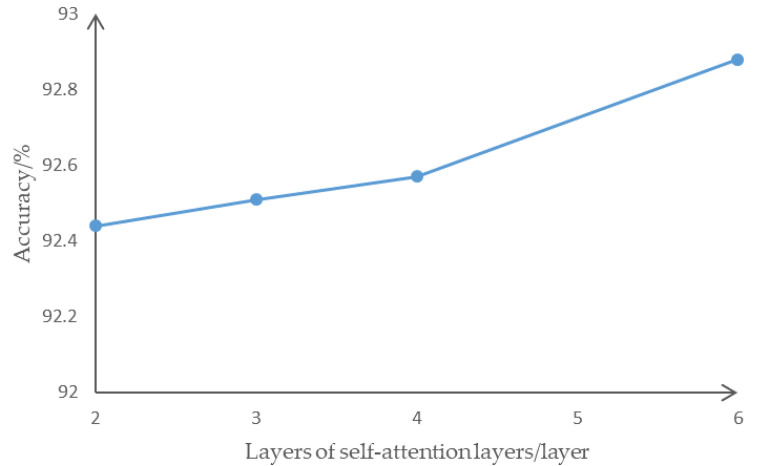
The prediction accuracy of different self-attention encoder layers.

The prediction accuracy first increased and then decreased with the number of self-attention heads and reached a peak at 6 heads of self-attention. When the multi-head self-attention exceeds 6 heads, too many independent features are extracted, making the information dispersion too high. Redundant independent feature extraction increases the original invisible connection in the data, and it is impossible to effectively obtain the interactive information that affects the intention of changing lanes. At the same time, the amount of calculation does not increase significantly as the number of self-attention heads increases, so this paper selects 6 self-attention heads for feature extraction. In the lane change intention data, in selecting the number of layers of the self-attention encoder, the prediction accuracy is slightly improved from layer 2 to layer 6, as shown in Figure 11.

The prediction accuracy increases with the increase of the number of self-attention layers. Each self-attention sub-layer realizes independent feature extraction and integration, gives extra attention to different timing information through sequential understanding, and obtains different features in end-to-end predictive decoding. However, the increase of the number of self-attention layers makes the number of backpropagation calculations significantly increase. Under the enormous computational cost, the prediction accuracy rate only increased from 92.4% to 92.8% in the coding from 2 to 6 layers. Therefore, a lightweight network with 2-layer coding is selected for prediction.

#### 4.2.2. Evaluation Parameter Settings

The evaluation indicators in this paper are multi-class accuracy, precision, recall, and F1 score, as well as TTE (Time To Event), as shown in Equations (14)–(18). The accuracy represents the proportion of the overall data predicted to be correct, the precision represents the proportion of the true positive in the predictions, and the recall represents the proportion of the true positive. The F1 score takes into account precision and recall. When the category proportions are not balanced, the precision, recall, and F1 score can evaluate the prediction results more comprehensively than the accuracy.

(14) $$Accuracy=\frac{TP+TN}{TP+TN+FP+TN},$$

(15) $$Precision=\frac{TP}{TP+FN},$$

(16) $$Recall=\frac{TP}{TP+TN},$$

(17) $$F1\;score=2\frac{P+R}{P\times R},$$

(18) $$TTE=t_{e}-t_{p},$$

where TP is the number of true positives, FP is the number of false positives, TN is the number of true negatives, FN is the number of false negatives, $t_{e}$ is the time when the vehicle right of way change occurs, and $t_{p}$ is the earliest time to predict the intention of the vehicle right of way change.

### 4.3. Results Analysis

There are 6380 frames with cut-in intention, 6780 frames with cut-out intention, and 15,930 frames with no-cut intention in the test set. The three-category behavior prediction results are shown in Table 2.

Through the ablation experiment, the addition of the DRAC increases the prediction accuracy, precision, recall, and F1 score by 2.8%, 2.9%, 2.6%, and 2.7%, respectively. The addition of the lane gap makes the prediction accuracy, precision, recall, and F1 score increase by 1.3%, 1.1%, 1.4%, 1.2%, respectively. Due to highway conditions, most of the forced lane changing stations are caused by vehicle driving conditions. The DRAC between vehicles can directly feedback the speed tolerance in the current lane and the target lane. Under loose road conditions, drivers tend to drive in lanes with greater speed tolerances. The lane gap can intuitively reflect the dense traffic of the current lane and the target lane. When the speed is allowed, the driver tends to drive in the sparse lane of the vehicle. Therefore, these two characteristics help the network predict vehicle interaction intentions better based on data.

**Figure 12:**
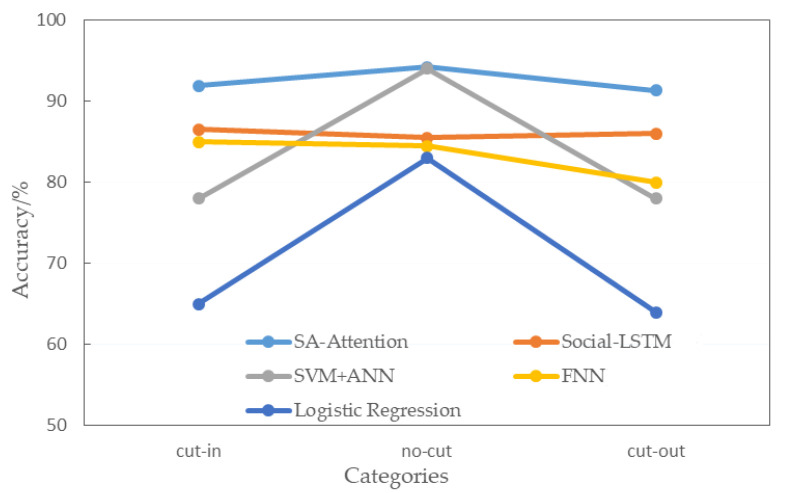
Comparison of prediction accuracy of different methods.

The method in this paper is compared with representative results from the same data set in recent years. The behavior prediction results are compared with the prediction accuracy of the feedforward neural network, logistic regression, Social-LSTM [33], and support vector machine + artificial neural network [34], as shown in Figure 12. The wathet line in the figure is the method in this paper.

As shown in Figure 12, the designed method in this paper has improved accuracy in all types of predictions. Notably, for cut-in and cut-out, two behavior that have a significant impact on the driving status of ego-vehicle, our model achieves the best performance compared to Social-LSTM and has increased by 6.2% and 6.9% respectively, and the overall prediction accuracy has improved significantly. In the multi-parameter evaluation system, it is compared with dynamic Bayesian networks [35], HSS based LSTM [36], attention-based LSTM [37], and Bayesian networks [38], as shown in Table 3.

**Figure 13:**
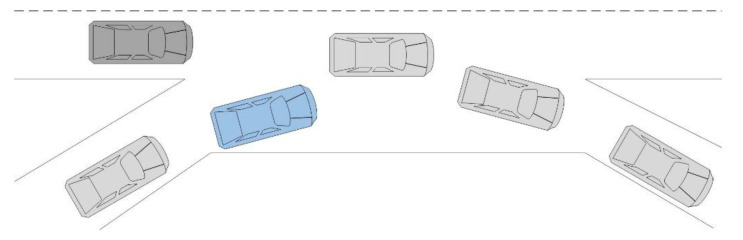
The interweaving area.

As shown in Table 3, compared with the dynamic Bayesian network, the F1 score, precision, and accuracy are increased by 16.2%, 31.2%, and 36.9%, respectively, and the recall is slightly reduced by 8.8%. Among them, the F1 score can more comprehensively reflect the prediction in the imbalanced category. This method is only 0.85 s shorter than the dynamic Bayesian network prediction time while improving the prediction effect. Under the premise of accurate prediction, the prediction time is guaranteed as much as possible. Compared with the LSTM network, the F1 score, precision and recall have increased by 6.5%, 4.6%, and 7.2%, respectively. The accuracy is slightly lower, but 1.4 s advance the prediction time on average. Compared with A-LSTM, F1 score, precision, recall, and accuracy are increased by 15.2%, 17.1%, 13.5%, and 15.6%, respectively, and the prediction time is extended by 1.702 s. Compared with the Bayesian network, F1 score, precision, recall, and accuracy are increased by 51.5%, 74.5%, 28.3%, and 62.1%, respectively. The prediction time is 1.87 s earlier, and the overall performance is greatly improved. By comparison, the advantages of the method proposed in this paper reflect that the time-series interactive information of the vehicle interactive intentions of the vehicle cluster can well reflect the vehicle state. Based on this information, the self-attention prediction method is superior to the vehicle’s interactive behavior intention prediction. This article is applied to two highways of US101 and I80 simultaneously. There are three road conditions: the buildup of congestion, the transition between uncongested and congested conditions and full congestion during the peak period. The congestion buildup period provides low speed and minor gap conditions to improve robustness. In the case of dense presence of vehicles, the prediction of the cut-out behavior of the front vehicle is helpful for the vehicle to maintain the current right-of-way and drive smoothly. The interweaving area (shown in Figure 13) and the main area have good generalization ability.

In the interweaving area, which is not considered in SA-LATM [33] and SVM + ANN [34], this paper realizes that it does not depend on the external environment information to effectively predict the interaction intentions of the surrounding vehicles to the ego-vehicle. The A-LSTM [37] uses the distance of the on-/off-ramp to solve the prediction in the interweaving area. However, there are various forms of access ramps in the expressway scene. The method in this paper, which is not based on map information, can adapt to different high-speed traffic scenes. Vehicles in the interweaving area are different from the vehicle cluster state in the traffic area due to the road itself. By exchanging information between the vehicle clusters, part of the state information can be interpretably classified. For example, when the vehicle entering the interweaving area is far away from the self-vehicle, it has a greater acceleration relative to the ego-vehicle. At the same time, when the relative acceleration is significant, it is still necessary to consider the DRAC, relative position, and lane clearance to the front and rear vehicles in the current lane. Through the interactive information between the vehicle clusters, the basis for the judgment of the right of way change under different road conditions is supplemented. In order that the abnormal part that affects the judgment of the interactive behavior does not affect the prediction result, the robust performance of the prediction under different road conditions is increased through feature extraction of the vehicle clusters.

## 5. Conclusions and Future Work

Prediction of vehicle interaction behavior is essential for planning and decision-making in autonomous driving vehicles. This paper proposes a target vehicle road-right change intention prediction method based on the interactive information of a five-vehicle cluster based on the ego-vehicle and the target vehicle. By extracting the interactive information of the target vehicle relative to the ego-vehicle, the interactive information of surrounding vehicles relative to the target vehicle, and the interactive information of vehicle clusters, a two-layer six-head self-attention encoder can be used to predict the intent of the target vehicle end-to-end. The Deceleration Rate to Avoid a Crash (DRAC) and the lane gap effectively complement the cluster information. Accuracy, precision, recall, and F1 score of the proposed model all exceeds 92% and time to event is up to 2.9s on the testing highway datasets, that includes three traffic flow situations and interweaving and main areas. Therefore, the model realizes the lane change prediction with class-imbalance data. In intelligent transportation, the intention prediction of target vehicles can effectively ensure the ride comfort of ego-vehicle, provide a basis and response time for decision-making and planning, and effectively reduce traffic accidents.

Since our proposed method is based on deep learning, there are some general limitations. First, the interpretability of deep learning is poor. It learns the implicit relationship between input and output features but not the causal relationship. Secondly, the neural network has many parameters. This paper uses edge calculation for a significant amount of time and relatively large computing power. Finally, the performance of deep-learning-based model dramatically relies on the collected data. In future research, we will consider more complicated urban traffic scenarios, including more intersections. Moreover, we will improve the interpretability of deep learning to fit more scenarios.

## Figures and Tables

**Table 1:** Interactive feature parameters.

Feature Type (Interaction Information)	Related Parameters	Parameter Representation
Between the target vehicle and the ego-vehicle	Relative speed/(m/s)	$v_{Tar}^{X}$ $,\;v_{Tar}^{Y}$
	Relative acceleration/(m/s^2^)	$a_{Tar}^{X}$ $,\;a_{Tar}^{Y}$
	Relative position/(m)	$x_{Tar}$ $,\;y_{Tar}$
Between surrounding vehicles and the target vehicle	Relative speed/(m/s)	$v_{f}^{X}$ $,\;v_{f}^{Y}$ $,\;v_{f1}^{X}$ $,\;v_{f1}^{Y}$ $,\;v_{r1}^{X}$ $,\;v_{r1}^{Y}$
	Relative acceleration/(m/s^2^)	$a_{f}^{X}$ $,\;a_{f}^{Y}$ $,\;a_{f1}^{X}$ $,\;a_{f1}^{Y}$ $,\;a_{r1}^{X}$ $,\;a_{r1}^{Y}$
	Lateral distance/(m)	$x_{cur-tar}$ $,\;x_{f-tar}$ $,\;x_{f1-tar}$ $,\;x_{r1-tar}$
	Deceleration rate to avoid a crash/(m/s^2^)	$DRAC_{cur}^{X}$ $,\;DRAC_{cur}^{Y}$ $,\;DRAC_{f}^{X}$ $,\;DRAC_{f}^{Y}$ $,\;DRAC_{f1}^{X}$ $,\;DRAC_{f1}^{Y}$ $,\;DRAC_{r1}^{X}$ $,\;DRAC_{r1}^{Y}$
Vehicle cluster	Relative position/(m)	$x_{tar}$ $,\;y_{tar}$ $,\;x_{f}$ $,\;y_{f}$ $,\;x_{f1}$ $,\;y_{f1}$ $,\;x_{r1}$ $,\;y_{r1}$
	Gap in the lane/(m)	$GAP_{cur}$ $,\;GAP_{tar}$

**Table 2:** Prediction results of VC-Attention.

**Intention**	**Precision**	**Recall**	**F1 Score**
Cut-in	0.907	0.932	0.921
Cut-out	0.912	0.916	0.910
No-cut	0.938	0.925	0.931

**Table 3:** Comparison of prediction and evaluation indicators by different methods.

**Method**	**VC-Attention**	**DBN [35]**	**HSS + LSTM [36]**	**A-LSTM [37]**	**BN [38]**
F1 Score	0.924	0.795	0.868	0.802	0.61
Precision	0.925	0.705	0.884	0.790	0.53
Recall	0.924	0.94	0.862	0.814	0.72
Accuracy	0.924	0.675	0.965	0.799	0.57
Time to event/s	2.9	3.75	1.5	1.198	1.03
